# Efficient Identification of the *MYC* Regulator with the Use of the CRISPR Library and Context-Matched Database Screenings

**DOI:** 10.3390/ijms23147723

**Published:** 2022-07-13

**Authors:** Yosuke Tanaka, Hidetaka Kambayashi, Akiko Yamamoto, Iichiroh Onishi, Keisuke Sugita, Miwa Matsumura, Sachiko Ishibashi, Masumi Ikeda, Kouhei Yamamoto, Masanobu Kitagawa, Morito Kurata

**Affiliations:** 1 Department of Comprehensive Pathology, Graduate School of Medicine, Tokyo Medical and Dental University, Tokyo 113-8510, Japan; tanaka.pth2@tmd.ac.jp (Y.T.); 170281ms@tmd.ac.jp (H.K.); akko-y@hotmail.co.jp (A.Y.); iichpth2@tmd.ac.jp (I.O.); keisuke.integralugita@gmail.com (K.S.); miwa.mw1116@gmail.com (M.M.); ishibashi.pth2@gmail.com (S.I.); mikepth2@tmd.ac.jp (M.I.); yamamoto.pth2@tmd.ac.jp (K.Y.); masa.pth2@tmd.ac.jp (M.K.); 2 Department of Obstetrics & Gynecology, Tokyo Medical University, Tokyo 160-8402, Japan

**Keywords:** CRISPR screening, promoter screening, *MYC*, database

## Abstract

*MYC* is a major oncogene that plays an important role in cell proliferation in human cancers. Therefore, the mechanism behind *MYC* regulation is a viable therapeutic target for the treatment of cancer. Comprehensive and efficient screening of *MYC* regulators is needed, and we had previously established a promoter screening system using fluorescent proteins and the CRISPR library. For the efficient identification of candidate genes, a database was used, for which mRNA expression was correlated with *MYC* using datasets featuring “Similar” and “Not exactly similar” contexts. *INTS14* and *ERI2* were identified using datasets featuring the “Similar” context group, and *INTS14* and *ERI2* were capable of enhancing *MYC* promoter activity. In further database analysis of human cancers, a higher expression of *MYC* mRNA was observed in the *INTS14* mRNA high-expressing prostate and liver cancers. The knockdown of *INTS14* in prostate cell lines resulted in decreased *MYC* mRNA and protein expression and also induced G0/1 arrest. This study confirmed that CRISPR screening combined with context-matched database screening is effective in identifying genes that regulate the *MYC* promoter. This method can be applied to other genes and is expected to be useful in identifying the regulators of other proto-oncogenes.

## 1. Introduction

The *MYC* family is composed of three genes: *MYCN*, *MYCL*, and *MYC* (*c-MYC*). *MYC* is one of the most widely studied human proto-oncogenes and is involved in the formation, maintenance, and progression of a very large number of human tumors [1,2,3,4].Despite much research on this important proto-oncogene, our understanding of the precise regulatory mechanisms underlying its function remains limited. In transcriptional regulation, bromodomain-containing 4 (BRD4) positively regulates the transcription of *MYC* by mobilizing positive transcription elongation factor b (P-TEFb), which phosphorylates the carboxy-terminal domain of RNA polymerase II (pol II) to hyperacetylated chromatin sites. This leads to the transcriptional elongation of pol II in the promoter proximal region of *MYC* [5]. Cyclin-dependent kinase 7 (CDK7) is a catalytic subunit of the transcription factor IIH complex and phosphorylate of the carboxy-terminal domain of Pol II; it is able to initiate *MYC* transcription [6,7,8,9]. Phosphorylation of eukaryotic translation initiation factor 4E binding protein 1 (4EBP1) is downstream of mammalian target of rapamycin (mTORC1) in the phosphatidylinositol 3-kinase (PI3K)/AKT/mTOR pathway. 4EBP1 promotes the translation of mRNAs of *MYC* containing long 5’-untranslated regions (5’-UTRs) with complex RNA secondary structures [10,11]. Cytoplasmic polyad-enylation element-binding protein (CPEB) binds to cytoplasmic polyadenylation elements containing UUUUAU or UUUUAAU sequences in the 3’-UTR of mRNA and negatively regulates the mRNA of *MYC* through interactions with Tob, an anti-proliferative protein [12,13].

Furthermore, the stability of the MYC protein is tightly regulated by the ubiquitin–proteasome system. Phosphorylation of the Thr58 residue of MYC proteins results in polyubiquitination of *MYC* by the E3 ligase F-box and WD repeat domain-containing 7 (FBW7), which is then degraded by the proteasome [14,15]. In this study, we focused on the inhibition of *MYC* promoter- and enhancer-mediated activation.

**Figure 1:**
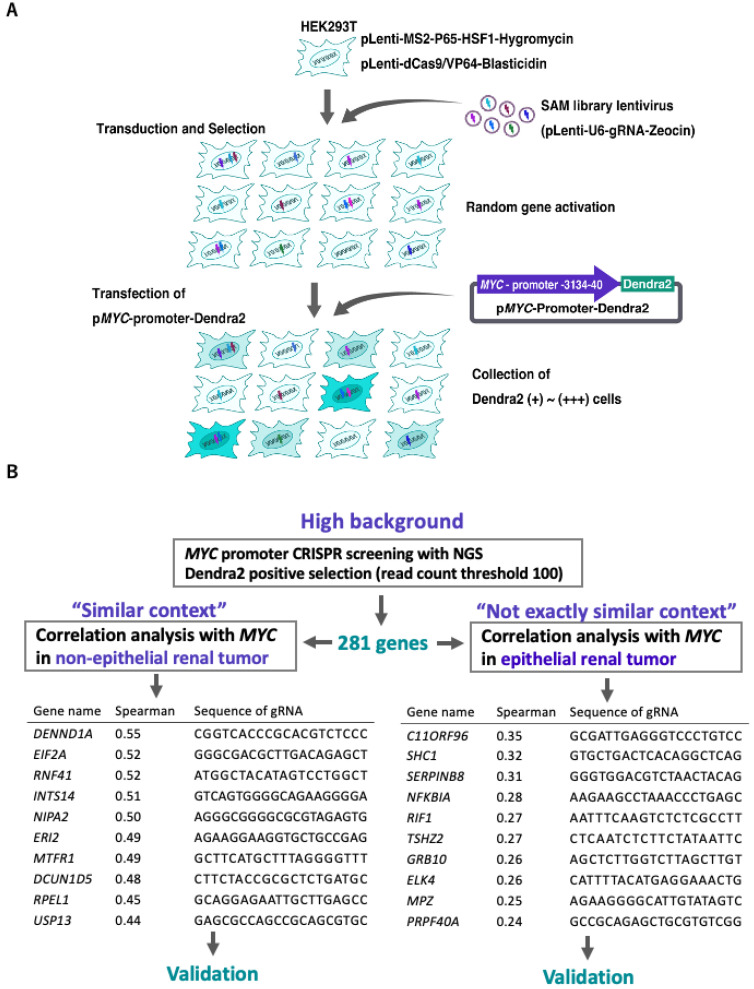
Modified *MYC* transcriptional activator screening and context-matched database screening. (**A**) Modified *MYC* transcriptional activator screening [16] (**B**) Schematic of the pipeline for narrowing down candidate genes using *MYC* promoter CRISPR screening and cancer databases.

To elucidate the mechanism regulating *MYC*, CRISPR screening is a powerful tool, and Yamamoto et al. (2020) previously established CRISPR activation screening with an *MYC* expression monitoring vector (pMYC-promoter-Dendra2), which incorporates a 3 kb *MYC* promoter region upstream of Dendra2, a fluorescent protein. Briefly, the CRISPR activation library and pMYC-promoter-Dendra2 were induced to HEK293T cells, and the Dendra2-positive cells with CRISPR random activations, which are supposed to be upregulated by *MYC*, were collected by a cell sorter. A total of 169 cells were collected, and 4 gRNA candidates were identified. However, only meiosis-1 associated protein (M1AP) gRNA-transfected cells showed *MYC* promoter activities. Although multiple genes were expected to be identified, only one clone and one gene, *M1AP*, were ultimately identified in the previous study [16]. During cell collection by flow cytometry, only cells with very high fluorescence intensity, which were thought to strongly increase *MYC* promoter activity, were isolated and collected to avoid false-positive cells, resulting in a strong expression of *MYC* that might be disadvantageous in handling harvested cells. To avoid this problem and to ensure comprehensive and efficient screening, we thought it necessary to collect more cells and obtain multiple candidate genes. Therefore, we collected a wide range of cells with mildly elevated fluorescence intensities, which were thought to have mildly elevated *MYC* promoter activity (Figure 1A). Because these cells are considered to contain a large number of background cells that are thought to have elevated fluorescence without elevated promoter activity and the number of candidate genes identified from the sampled cells was very large, it was inefficient and difficult to validate them all in detail. Therefore, we concluded that it would be effective to narrow down the candidate genes using the Cancer Genome Database. 

Several studies have shown that the context of the cell line influences the experimental results, and in this study, we focused on the cell context to optimally narrow the number of candidate genes. Kulkarni et al. (2016) examined mechanistic differences in the regulation of a let-7a-targeted (wild-type) or resistant (mutant) engineered Renilla transcript across various mammalian cell lines of diverse origin and found that the strength of miRNA regulation varied across the cell lines [17]. No prior study has used a database in addition to CRISPR screening to narrow down candidate genes, along with the “context” of the cells used in the experiment. In the present study, we obtained a sufficient number of candidate genes for comprehensive refinement and finally identified *Integrator Complex Subunit 14* (*INTS14*) as an *MYC* regulator.

## 2. Results

### 2.1. High-Throughput Gene Purification to Narrow down Candidate Genes

**Figure 2:**
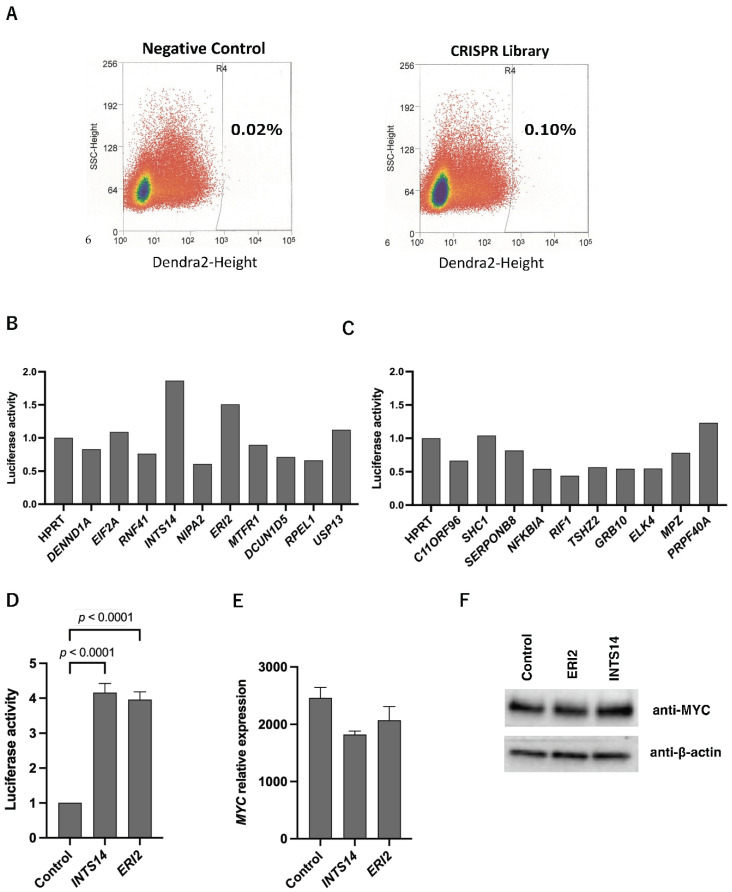
*MYC* promoter activity of candidate genes in a “Similar” and a “Not exactly similar” context. (**A**) *MYC* promoter CRISPR screening using FACS. The weakly and strongly Dendra2-positive cells in which the *MYC* promoter is thought to be active were sorted and collected. The negative control was HEK293T cells without the CRISPR activation library. (**B**) The top 10 genes in a “Similar” (pediatric rhabdoid tumor) context that correlated strongly with *MYC* in terms of mRNA expression were CRISPR-activated in HEK293T cells. After 48 h, luciferase activity was evaluated using Dual-Luciferase Reporter Assay. (**C**) The top 10 genes in a “Not exactly similar” (clear cell renal cell carcinoma) context that correlated strongly with *MYC* in terms of mRNA expression were CRISPR-activated in HEK293T cells. After 48 h, a Dual-Luciferase Reporter Assay was performed. (**D**) *INTS14* or *ERI2* was overexpressed using the CAG promoter in HEK293T cells. After 48 h, the results were evaluated using a Dual-Luciferase Reporter Assay. (**E**) *INTS14* or *ERI2* was overexpressed using the CAG promoter in HEK293T cells. After 48 h, the results were evaluated by qPCR. (**F**) *INTS14* or *ERI2* was overexpressed using the CAG promoter in HEK293T cells. After 48 h, the results were evaluated by western blotting. (**D**,**E**) Control: GFP was overexpressed with a CAG promoter. The values shown are the means ± standard error (SEM) (*n* = 3).

In the present study, to obtain sufficient candidate genes through the comprehensive analysis of *MYC* promoter region regulation, cells with weakly positive fluorescence intensity, which were thought to be borderline regions with mildly elevated promoter activity, were also collected. However, there may be many cells associated with background cells that are not fluorescence enhanced by the CRISPR library, so we concluded that efficient screening would be possible by combining them with expression analysis using the database (Figure 1B). The number of high-to-border Dendra2 expression cells was 0.10% of the total number of cells. The same gating for cells without lentiSAMv2 included 0.02% of all cells (Figure 2A). Genomic DNA was extracted from the collected cells, and the gRNA-containing region was amplified by polymerase chain reaction (PCR). The PCR products were applied to the NGS. Read counts of gRNAs with NGS were set as the threshold for 100 counts. Eventually, 281 candidate gRNAs were obtained. Using the cBioPortal for Cancer Genomics database, the correlation between *MYC* expression and candidate genes was examined. In addition, as a “Similar” context, we selected the Pediatric Rhabdoid Tumor (TARGET, 2018) dataset; rhabdoid tumors are relatively similar to HEK293T cells in terms of pediatric kidney mesenchymal cells. As a “Not exactly similar” context, we selected the Clear Cell Renal Cell Carcinoma (TCGA, PanCancer Atlas) dataset; renal cell carcinoma is similar to HEK293T cells in that they are kidney-derived cells but different in that they are epithelial. The top 50 candidate genes that correlated strongly with *MYC* in terms of mRNA expression in both cases were listed (Table S1A,B). In pediatric rhabdoid tumors, the top 10 gene names (and the Spearman’s rank correlation coefficients) with the strongest *MYC* correlation for mRNA expression (“Similar” context group) were *DENND1A* (r = 0.54711568), *EIF2A* (r = 0.5243129), *RNF41* (r = 0.52159468), *INTS14* (r = 0.51162791), *NIPA2* (r = 0.50181214), *ERI2* (r = 0.49154334), *MTFR1* (r = 0.48791906), *DCUN1D5* (r = 0.48489882), *RPEL1* (r = 0.44708725), and *USP13* (0.44201148). In clear cell renal cell carcinoma, the top 10 gene names (and the Spearman’s rank correlation coefficients) with the strongest *MYC* correlation for mRNA expression (“Not exactly similar” context group) were *C11ORF96* (r = 0.34183769), *SHC1* (r = 0.31280309), *SERPINB8* (r = 0.30896319), *NFKBIA* (r = 0.27450634), *RIF1* (r = 0.26827247), *TSHZ2* (r = 0.26435419), *GRB10* (r = 0.2582793), *ELK4* (r = 0.25595022), *MPZ* (r = 0.24417635), and *PRPF40A* (r = 0.23916876) (Figure 1B). 

### 2.2. INTS14 and ERI2 Identified as Candidates for the MYC Regulator with Context-Matched Database Screening

To verify the effects of candidate genes on *MYC* promoter activity, expression induction was tested in the “Similar” and “Not exactly similar” context groups using the CRISPR activation system. In the “Similar” context group, the luciferase activity of *INTS14* was 1.87-fold higher than that of *HPRT* activation, and the *exoribonuclease family member 2* (*ERI2*) luciferase activity was 1.51-fold higher than that of *HPRT* luciferase activity. These two genes were considered promising genes for increasing *MYC* transcriptional activity (2/10) (Figure 2B). There were no activity candidate genes in the “Not exactly similar” context group (0/10) (Figure 2C). For further validation of *INTS14* and *ERI2*, two genes were overexpressed; pT3.5-CAG-*INTS14* or pT3.5-CAG-*ERI2* were transfected into HEK293T. Luciferase activity was 4.16-fold in *INTS14*-overexpressing cells and 4.00-fold in *ERI2*-overexpressing cells compared to the control vectors (Figure 2D). The mRNA of HEK293T with pT3.5-CAG-*INTS14* or pT3.5-CAG-*ERI2* was harvested, and quantitative PCR (qPCR) was performed. Contrary to expectations, no increase in the expression of *MYC* mRNA was observed in either *INTS14*- or *ER*I*2*-overexpressing HEK293T cells (Figure 2E). Western blotting of HEK293T cells also showed no significant enhancement in the protein expression of either *INTS14*- or *ERI2*-overexpressing cells (Figure 2F and Figure S1A,B). The knockdown of *INTS14* or *ERI2* with short interference RNAs (siRNAs) in HEK293T cells was performed, and qPCR showed no decrease in *MYC* mRNA in either *INTS14* or *ERI* knockdown HEK293T cells (Figure S1C,D). In HEK293T cells, *INTS14* is an active *MYC* promoter, but the changes could not be observed as an increase or decrease in *MYC* mRNA. We concluded that additional regulatory mechanisms of *MYC* expression could be involved.

### 2.3. High MYC Expression in Tumors Associated with the High Expression of INTS14

**Figure 3:**
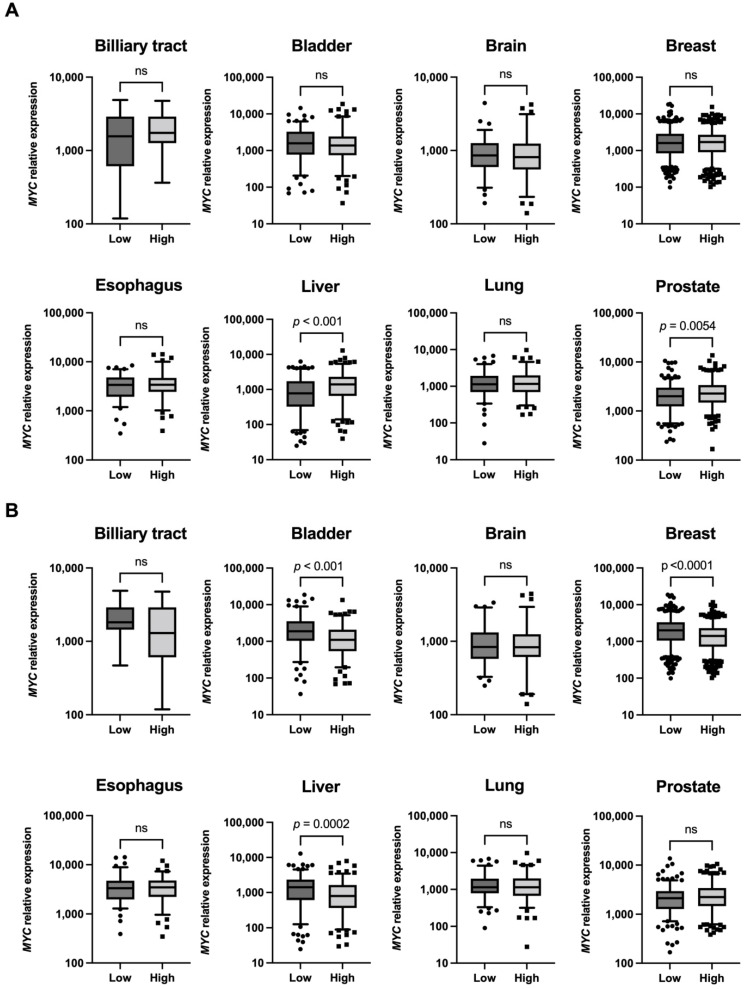
*MYC* mRNA expression was higher in the groups of tumors with high *INTS14* mRNA expression. (**A**) The cases were divided into two groups, low and high, based on the median *INTS14* mRNA expression level. The Mann–Whitney U test was performed for each *MYC* mRNA expression level. (**B**) *ERI2* and *MYC* mRNA were compared, as in (**A**). Not significant (ns).

To search for tumors in which regulation by the *MYC* promoter may be detectable as changes in mRNA abundance, we used cBioPortal for Cancer Genomics. The Mann–Whitney U test was used to compare the mRNA expression relationship between *MYC* and *INTS14* or *ERI2* in a dataset featuring each tumor type. The datasets selected included data on tumors of major organs for which information on mRNA expression levels existed. In the Prostate Adenocarcinoma (TCGA, Firehose Legacy) and Liver Hepatocellular Carcinoma (TCGA, PanCancer Atlas) datasets, *MYC* mRNA was highly expressed in the *INTS14* mRNA high-expression group (Figure 3A). There was no dataset on tumor types with a significantly higher expression of *MYC* mRNA in the *ERI2* mRNA high-expressing group (Figure 3B). 

### 2.4. The Knockdown of INTS14 Decreases MYC Expression in the Prostate Cancer Cell Line

**Figure 4:**
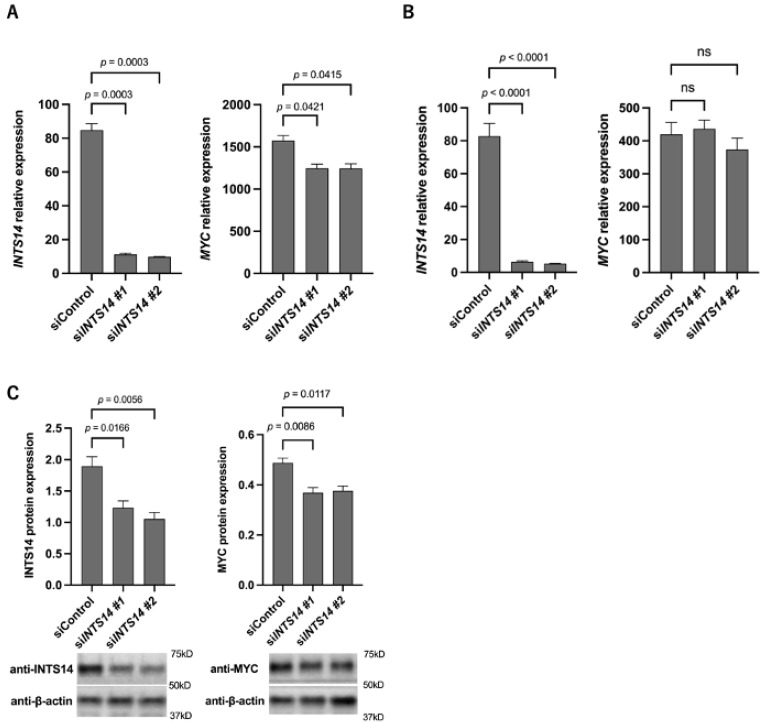
Knockdown of *INTS14* reduces *MYC* mRNA and protein expression in prostatic cell lines (PC-3). (**A**) qPCR of *INTS14* knockdown by siRNA transfection in PC-3 cells. (**B**) qPCR of *INTS14* knockdown by siRNA transfection in HuH-7 cells. (**C**) Western blotting analysis of INTS14 knockdown by siRNA transfection in PC-3 cells. The values shown are the means ± SEM (*n* = 3). Not significant (ns).

*MYC* mRNA was highly expressed in the *INTS14* mRNA high-expression group in prostate and liver cancer cell lines in the database, PC-3 cells (prostate cancer-derived cells) and HuH-7 cells (liver cancer-derived cells) were utilized for the following experiments. *INTS14* siRNA was transfected into PC-3 or HuH-7 cells, and the cells were harvested after 48 h. The qPCR validated the decreased expression of *MYC* mRNA in transfected PC-3 cells, and a decreased expression of *INTS14* mRNA was confirmed (Figure 4A). A qPCR evaluation of HuH-7 cells showed no significant decrease in *MYC* mRNA for *INTS14* siRNA #1 or #2 (Figure 4B). The results suggested that *INTS14* may regulate *MYC* mRNA in PC-3 cells, and further experiments were performed. Western blotting showed a decrease in protein expression (Figure 4C). 

### 2.5. Knockdown of INTS14 Induces G0/1 Arrest in Prostate Cancer Cells

**Figure 5:**
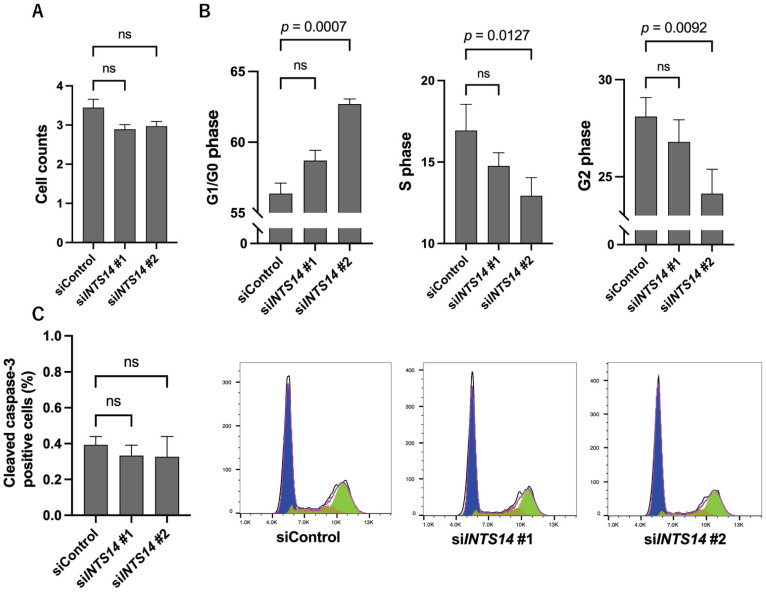
Knockdown of *INTS14* causes G0/1 arrest in PC-3 cells. (**A**) PC-3 cell count following *INTS14* knockdown by siRNA transfection. (**B**) Cell cycle assay of *INTS14* knockdown by siRNA transfection in PC-3 cells. (**C**) Apoptosis analysis of *INTS14* knockdown by siRNA transfection in PC-3 cells. The values shown are the means ± SEM (*n* = 3). Not significant (ns).

Despite a decrease in the expression of MYC at the protein level, the cell numbers showed a slight but insignificant decrease (Figure 5A). To observe the detailed effects of *INTS14* knockdown on the cell cycle and apoptosis in PC-3 cells, *INTS14* siRNAs were transfected to HEK293T and harvested 48 h later. Cell cycle analysis showed a trend toward G0/1 arrest with an increased G0/G1 phase and decreased S and G2 phases for both *INTS14* siRNA #1 and #2 knockdown. *INTS14* siRNA #2 showed a significant difference (Figure 5B and Figure S2A). No increase in the number of cells positive for cleaved caspase-3 or annexin V was observed, and no increase in apoptosis was observed (Figure 5C and Figure S2B).

## 3. Discussion

Yamamoto et al. (2020) identified an *MYC* promoter regulator using the CRISPR library and the “*MYC* expression monitoring vector.” In a previous study, they sorted the cells with elevated promoter activity using CRISPR screening with a strict threshold to prevent the collection of background cells as much as possible [16]. During the CRISPR screening, the HEK293T cells collected in the sorting represented 0.10% of the total number of cells, whereas the same gating for cells without lentiSAMv2 (negative control) included 0.02% of the total HEK293T cells, suggesting that at least 20% of the cells corresponded to the background. By optimally narrowing down this background-rich gene list using the cBioPortal database, the *MYC* promoter regulators *INTS14* and *ERI2* were identified. In the refinement, we divided the list into two groups—”Similar” context, which is as similar as possible, and “Not exactly similar” context, which is similar but partially different—to narrow down the list of candidate genes that are strongly correlated with *MYC* in terms of mRNA expression. *INTS14*, which was included in the “Similar” context group, showed increased *MYC* promoter activity when induced or overexpressed in HEK293, but there was no increase in *MYC* mRNA. In short, *INTS14* regulates the *MYC* promoter in HEK293T, but the change cannot be observed as an increase or decrease in *MYC* mRNA, suggesting that mRNA regulation may strongly occur post-transcriptionally. Takwi et al. (2021) screened miRNAs for their ability to regulate *MYC* functions and directly target the *MYC* 30UTR using a reporter assay and identified miR-33b as a negative regulator of *MYC* [18]. We considered the possibility that the reason for the lack of change in mRNA levels in this study may be due to other regulatory factors. 

INTS14 is one of the subunits of the Integrator complex, which mediates the 3′-end processing of small nuclear RNA (snRNA). snRNA is a component of the spliceosome required for the splicing of pre-mRNA and for the expression of protein-coding genes [19,20,21,22,23]. Sabath et al. identified a new module of INT, INTS10-INTS13-INTS14, by copurification and coprecipitation, and the INTS10-INTS13-INTS14 module was found to bind to the INTS4-INTS9-INTS11 module (cleavage module). Furthermore, they implied that although the INTS10-INTS13-INTS14 module itself is not as central to snRNA processing as INTS11, the inhibition of the INTS10-INTS13-INTS14 module causes inappropriate INT assembly due to a disruption of its binding to the cleavage module, causing reduced efficiency of snRNA maturation [24,25]. In the present study, the *MYC* mRNA level was decreased by the knockdown of *INTS14*. The knockdown of *INTS14* might prevent the binding of INTS10-INTS13-INTS14 to the cleavage module, and the inhibition of snRNA processing might have resulted in the inhibition of pre-mRNA splicing, which was then observed as a decrease in *MYC* mRNA. Indeed, Ruan et al. suggested that ubiquitin-specific peptidase 39 with the spliceosome including snRNA could contribute to *MYC* mRNA upregulation [26]. The knockdown of *INTS14* in PC-3 resulted in a decrease in *MYC* mRNA and protein expression, as well as G0/1 arrest. These results suggest that *INTS14* regulates the *MYC* promoter, which were observed as changes in *MYC* mRNA levels, protein expression levels, and cell cycles in PC-3. The knockdown of *INTS14* by siRNA introduction tended to induce G0/1 arrest, while *INTS14* siRNA #1 had a slightly weaker effect; however, no statistically significant difference was found. Four splice variants were identified in *INTS14*, which may have affected the strength of the siRNA effect.

In addition to *MYC* promoter screening using the CRISPR library, we narrowed down the candidate genes with a strong correlation to *MYC* in terms of mRNA expression using the “Similar” and a “Not exactly similar” contexts, respectively, and then validated that *INTS14* and *ERI2* were identified as *MYC* promoter activators. We compared the mRNA expression relationship between *MYC* and *INTS14* or *ERI2* using the database again to identify tumors in which regulation by *MYC* promoters could alter mRNA levels, and we used cell lines with similar contexts in our experiments. *INTS14* was then identified as an activator of *MYC* mRNA expression, establishing that this method is useful for identifying *MYC* transcription factors. This method is expected to be useful in identifying the genes that activate *MYC*, thus allowing the identification of regulatory factors of other proto-oncogenes, as well as in facilitating drug discovery against other malignant tumors driven by oncogenes.

## 4. Materials and Methods

### 4.1. Cell Culture

Human embryonic kidney cells (HEK293T) were purchased from the Japanese Cancer Research Bank and maintained in Dulbecco’s Modified Eagle Medium (DMEM, Fuji Film) with 10% fetal bovine serum (Gibco) and penicillin/streptomycin (Gibco) in a 5% CO_2_ atmosphere at 37 °C. Human prostate carcinoma cells (PC-3) were purchased from the RIKEN BRC cell bank and maintained in Roswell Park Memorial Institute’s medium (RPMI-1640, Fuji Film) with 10% fetal bovine serum and penicillin/streptomycin with 5% CO_2_ at 37 °C. Human hepatoma cells (HuH-7) were purchased from the Japanese Cancer Research Bank and maintained in DMEM (Fuji Film) with 10% fetal bovine serum and penicillin/streptomycin with 5% CO_2_ at 37 °C.

### 4.2. SAM Library Screening

HEK293T cells (5 × 10^5^ cells/well) were seeded in 6-well cell culture plates 1 day prior to transfection. The next day, 3 μg of lentiviral plasmid was transfected along with 1 μg of pMD2.G and 2 μg of pCMV using the Lipofectamine 3000 reagent (Invitrogen). Then, 12 h after transfection, the medium was replaced with fresh DMEM. Two days after transfection, the viral supernatant was collected and filtered through Millex-HP 0.45 μm (Millipore). One day prior to transfection, the target HEK293T cells (5 × 10^5^ cells/well) for lentivirus infection were seeded in 6-well plates and transduced with 5 μg/mL of Polybrene (Sigma) to this lentiviral supernatant. The lentiviral plasmids used were lentiMPHv2 (Addgene, #89308), lentidCAS-VP64-blast (Addgene, #61425), and lentiSAMv2 (Addgene, #61597). LentiMPHv2 and lentidCAS9-VP64 were transduced into HEK293T cells and treated with hygromycin B or blasticidin, respectively, for 2 weeks. Control guide RNA (gRNA) (hypoxanthine-guanine phosphoribosyltransferase; HPRT), *MYC*-activated gRNA, and lentiSAMv2, a CRISPR activation library, were transduced into HEK293T cells expressing MPH-dCas9-VP64 and treated with Zeocin (300 μg/mL, Invivogen) for 2 weeks. The sequence of the control gRNA (HPRT) was AGCTAGAGTGCTCGGCTGCC. HEK293T cells expressing MPH-dCas9-VP64-SAMv2, which were treated with Lipofectamine 3000 reagent (Invitrogen), were used to introduce a reporter system (pMYC-promoter-Dendra2) with Dendra2 and a −3.1 kb *MYC* promoter region. After 72 h, the cells were collected and separated using a cell sorter. Genomic DNA was harvested, and PCR was performed on the gRNA-containing region using KOD-FX (Toyobo). The sequences of the PCR primers were as follows: U6 F-NGS—TCGTCGGCAGCGTCAGATGTGTATAAGAGACAGtcttgtggaaaggacgaaacaccg and EF1a139R NGS—GTCTCGTGGGCTCGGAGATGTGTATAAGAGACAGggagccagtacgacatca. The gRNA sequences were determined using the Illumina NovaSeq 6000 platform. Cutadapt was applied to trim adapter sequences, and Trimmomatic was used to remove regions with low quality scores (Hokkaido System Science CO, Ltd.) [27]. A read count of 100 was set as the threshold, and 281 candidate guide RNA sequences were obtained.

### 4.3. Plasmids

Double-stranded DNA fragments of *INTS14* and *ERI2* cording regions with attB sites were purchased from gBlocks (IDT) and incorporated into the pENTR221 vector using the Gateway BP clonase. The DNA fragments were then transferred into the pT3.5-CAG-DEST vector using Gateway LR clonase. pT3.5-CAG-*INTS14,* or pT3.5-CAG-*ERI2* were co-transfected along with the pGL4-*MYC* promoter luciferase reporter vector, pRLSV luciferase reporter vector, and SBI super PB (SBI) to the HEK293T cells. Samples were taken 48 h after transfection with the Lipofectamine 3000 reagent (Invitrogen).

### 4.4. Reporter Assay

The luciferase reporter vector incorporating fragments of the *MYC* promoter into the pGL4 vector (Promega) was utilized [16]. The CRISPR activation system (plenti-dCas9-VP64, plenti-MS2-p65-HSF1, and pE1-U6-gRNA-MS2 [28] of the individual candidate genes) or pT3.5 overexpression vectors were transfected to HEK293T cells expressing the luciferase reporter vector pRL Renilla using the Lipofectamine 3000 reagent (Invitrogen). Cells were collected 48 h after transduction. Luciferase activity was measured by following the protocol of the dual-luciferase reporter assay system (Promega) and Lumat LB9507 (Perkin Elmer). The luciferase activity values were standardized with the luciferase activity value of pE1-h-HPRT. 

### 4.5. Database Analysis

The cBioPortal for Cancer Genomics [29,30] was used to analyze the expression levels of *INTS14* mRNA and *MYC* mRNA or *ERI2* mRNA and *MYC* mRNAs in each of the following databases: Prostate Adenocarcinoma (TCGA, Firehose Legacy), Liver Hepatocellular Carcinoma (TCGA, PanCancer Atlas), Lung Adenocarcinoma (TCGA, Firehose Legacy), Breast Invasive Carcinoma (TCGA, Firehose Legacy), Bladder Cancer (MSK/TCGA, 2020), Cholangiocarcinoma (TCGA, PanCancer Atlas), Esophageal Adenocarcinoma (TCGA, PanCancer Atlas), and Glioblastoma Multiforme (TCGA, PanCancer Atlas). The Mann–Whitney U test was performed for *MYC* mRNA expression levels in the two groups by dividing the selected dataset based on the median values of *INTS14* or *ERI2* mRNA expression levels.

### 4.6. Short Interference RNAs

siRNAs of *INTS14* or *ERI2* were introduced to HEK293T cells and HuH-7 or PC-3 using the Lipofectamine 3000 reagent (Invitrogen). Silencer Select Negative Control No.1 siRNA (Thermo Fisher Scientific; no.4390843) was used. The sequences of siRNA (sense strand) were *INTS14* #1: GGCAGAUUUUUACUAUUGA, *INTS14* #2: GAAUGGUAGCGAUUGUUCA, *ERI2* #1: GGAGUAUGAGUGUAAAAGA, and *ERI2* #2: GGACGAUUCUCGGAAUACU and were purchased from BEX CO., LTD. After 48 h of transduction, inducted cells were collected, and RNA was isolated using the RNeasy Mini Kit (Qiagen) according to the manufacturer’s instructions. The ReverTra AceR qPCR RT Master Mix (Toyobo) was used to generate complementary DNA. β-actin was used as an endogenous control. A qPCR analysis was performed on an ABI Prism 7900HT (Applied Biosystems) with SYBR Mix (QIAGEN). The sequences of PCR primers used for gene expression were as follows: *MYC*—5′-CGACTCTGAGGAGGAACAAGAA-3′ (forward) and 5′-CAGCAGAAGGTGATCCAGACT-3′ (reverse), β-actin—5′-CACAGCCTCGCCTTTGCC-3′ (forward) and 5′-CACAGCCTCGCCTTTGCC-3′ (reverse), *INTS14*—5′-CAATTCCCTTGCCAGGTTGTC-3′ (forward) and 5′-TGAGTGGCTAGGGAATGTCC-3′ (reverse), and *ERI2*—5′-AGGCATAAAGCAGGCTCAAG-3′ (forward) and 5′-GTTGCTGAATCTTATGAATCCATTT-3′ (reverse). The ΔΔCt method using β-actin was implemented to calculate the mRNA levels of the target genes. 

### 4.7. Western Blots

Cells collected for western blotting were lysed in a sodium dodecyl sulfate (SDS) buffer containing 25% 0.125 M Tris-HCl (pH 6.8), 20% glycerol, 4% SDS, and 10% 2-mercaptoethanol with bromophenol blue, and DNA was disrupted by sonication on ice. Samples were separated on 4–20% SDS-polyacrylamide gel electrophoresis (SDS-PAGE) gels (Bio-Rad) and electro-transferred to Immobilon polyvinylidene difluoride (PVDF) membranes (Millipore). The membranes were incubated in Bullet Blocking One (Nacalai Tesque) for 5 min at room temperature. Next, the membrane was incubated with the primary antibodies anti-INTS14 (Atlas Antibodies, HPA040255), anti-c-Myc (Abcam ab32072), or anti-β-actin (Cell Signaling Technology, #4967) overnight at 4℃, all at a dilution of 1:1000. After washing with TBS-T, the membrane was incubated with the second antibody, horseradish peroxidase (HRP)-labeled anti-rabbit IgG (GE Healthcare, NA934), for 1 h at room temperature at a dilution of 1:5000. Protein bands were visualized using the Clarity Western ECL Substrate (Bio-Rad).

### 4.8. Cell Cycle and Apoptosis Analysis

For cell cycle analysis, the collected cells were treated with 0.1% TRITON-X. The cells were mixed thoroughly by gentle inversion, allowed to stand for 2 min on ice, treated with RNase at a concentration of 40 μg/mL, and allowed to stand for 10 min at room temperature. Cells were stained with propidium iodide (Sigma Aldrich) at a concentration of 25 μg/mL, and propidium iodide was measured by BD FACS (Becton Dickinson). For the apoptosis analysis, the collected cells were resuspended in 4% formaldehyde, fixed for 15 min at room temperature, and then permeabilized with 90% cold methanol for 10 min on ice. The anti-cleaved caspase-3 antibody (cell signaling, #9661) at a dilution of 1:800 was incubated for 1 h at room temperature. Cells were treated with a secondary antibody (Anti-Rabbit IgG (PE Conjugate); Cell Signaling) solution at a dilution of 1:500 at room temperature for 30 m. The fluorescence intensity of PE was measured using BD FACS.

### 4.9. Statistical Analysis

The data were statistically analyzed using GraphPad Prism Version 9.3.1 (350) software. Three or more groups were compared for mean values using a one-way analysis of variance (ANOVA) test. The mRNA expression levels of MYC and INTS14 or ERI2 were compared using the Mann–Whitney U test. A value of p < 0.05 was considered statistically significant for all analyses.

## Data Availability

Not applicable.

## Supplementary Materials

The following supporting information can be downloaded at: https://www.mdpi.com/article/10.3390/ijms23147723/s1.
