# Supplementary material for: Efficient Identification of the MYC Regulator with the Use of the CRISPR Library and Context-Matched Database Screenings
Source: Int J Mol Sci. 2022 Jul 13;23(14):7723. doi: 10.3390/ijms23147723 (PMC9317319; doi:10.3390/ijms23147723)
Supplement: Supplementary file 1 [file ijms-23-07723-s001.zip › ijms-1773840-supplementary.pdf]

## Supplementary Figure S1

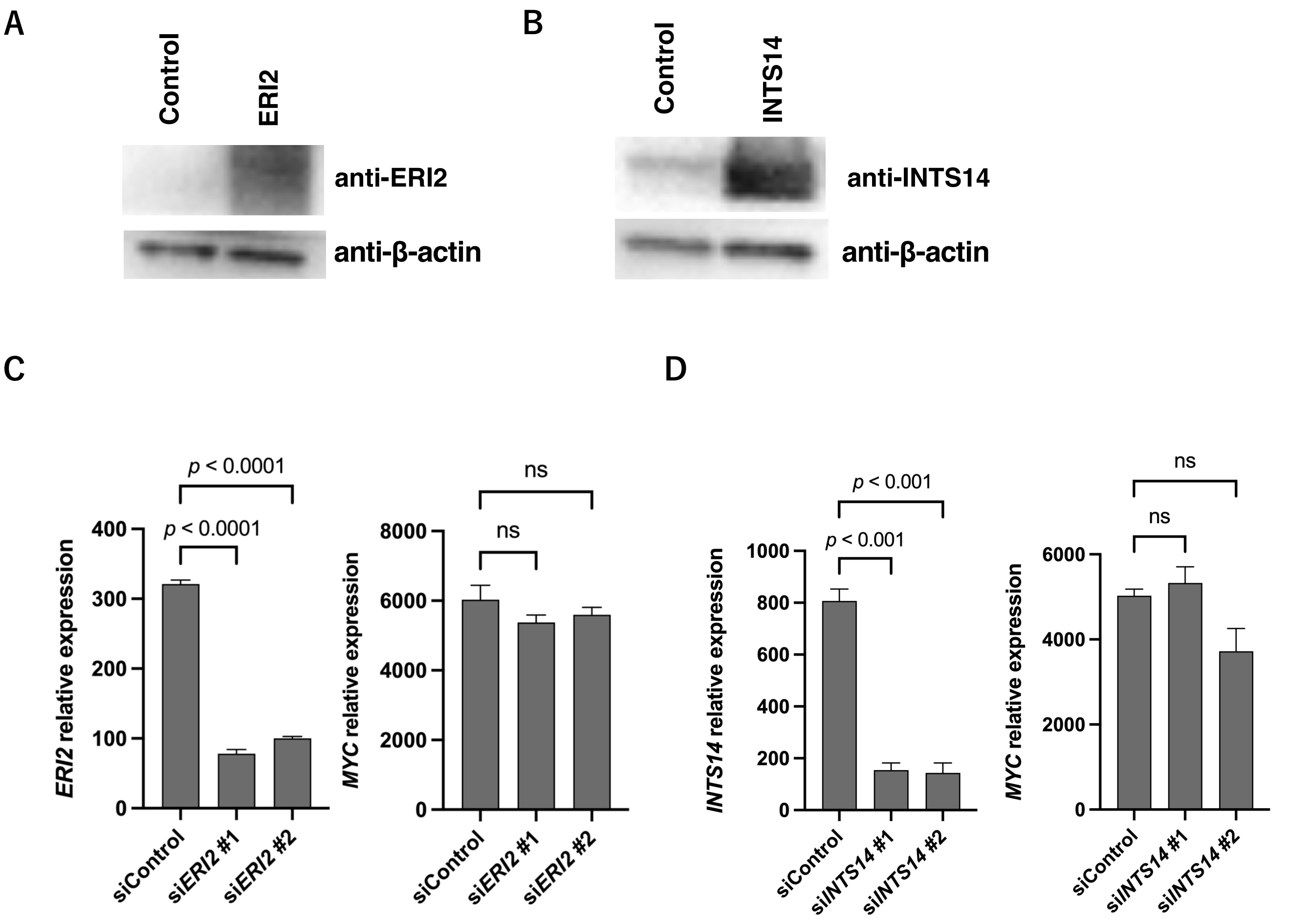


**Supplementary figure S1**

A,B. ERI2 or INTS14 were overexpressed using the CAG promoter in HEK293T cells. After 48 h, the results were evaluated by western blotting. Control: GFP was overexpressed with CAG promoter.

C. qPCR after 48 h of *ERI2* knockdown by siRNA transfection in HEK293T cells.

D. qPCR after 48 h of *INTS14* knockdown by siRNA transfection in HEK293T cells.

The values shown are means ± SEM (n = 3).

## Supplementary Figure S2

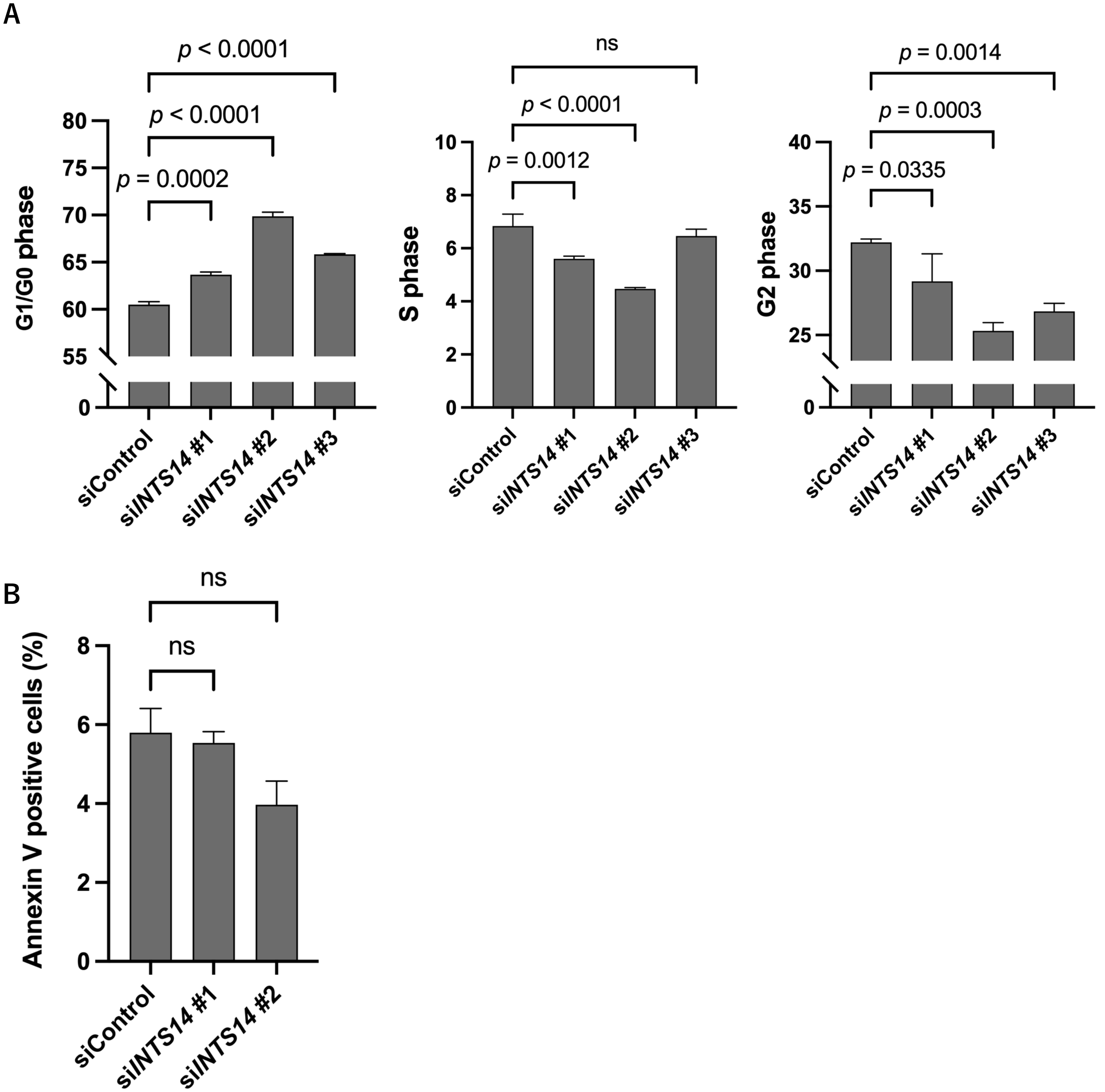


**Supplementary figure S2**

A. Cell cycle assay of *INTS14* knock down by siRNA transfection in PC-3 cells. The sequences of siRNA (sense strand) were *INTS14* #1: GGCAGAUUUUUACUAUUGA, *INTS14* #2: GAAUGGUAGCGAUUGUUCA, *INTS14* #3: GAGAGAUUAUAAUACCCUA and were purchased from BEX CO., LTD.

B. Apoptosis analysis using Annexin V of *INTS14* knockdown by siRNA transfection in PC-3 cells. The values shown are means ± SEM (n = 3).

Material and Methods

Apoptosis analysis using Annexin V was performed following the protocol of the Annexin V-FITC (Reagent) (MEDICAL ＆ BIOLOGICAL LABORATORIES CO., LTD.) and annexin V was measured by BD FACS (Becton Dickinson).
